# Nepotistic colony fission in dense colony aggregations of an Australian paper wasp

**DOI:** 10.1038/s41598-022-17117-y

**Published:** 2022-07-27

**Authors:** Koji Tsuchida, Norio Ishiguro, Fuki Saito-Morooka, Jun-ichi Kojima, Philip Spradbery

**Affiliations:** 1 Laboratory of Insect Ecology, Faculty of Applied Biological Sciences, Gifu University, Yanagido 1-1, Gifu, 501-1193 Japan; 2 Australian National Insect Collection, CSIRO, Canberra, ACT 2601 Australia; 3 School of Biological Sciences, Flinders University, Adelaide, SA 5001 Australia; 4 Natural History Laboratory, Faculty of Science, Ibaraki University, Mito, Japan; 5 XCS Consulting Pty Ltd, Yarralumla, ACT 2600 Australia

**Keywords:** Ecology, Evolution, Zoology

## Abstract

Social insects are highly diverse in their social structures, aside from the consistent presence of reproductive castes. Among social insects, the Australian paper wasp *Ropalidia plebeiana* constructs extremely dense colony aggregations consisting of hundreds of colonies within a few square meters; however, little is known about the aggregation structures. We genetically analyzed the colony and population structure of *R. plebeiana*, and concomitant variations in colony sex ratios. In spring, the foundress (candidate queen) group started their colonies on a single old comb from the previous season, subsequently dividing these old combs via relatedness-based comb-cutting. Female philopatry, a prerequisite condition of Local Resource Competition (LRC), was confirmed. The colony sex ratio of reproductive individuals (male and female offspring for the next generation) became slightly male-biased in larger colonies, as predicted under LRC. However, the number of foundresses was positively associated with the number of reproductive individuals, suggesting that Local Resource Enhancement (LRE) also operates. Although the population structure appears to meet the prerequisites of LRC, the sex ratio appears to be modulated by factors other than LRC. Rather, through LRE, the availability of female helpers at the founding stage is likely to mitigate the sex ratios predicted under LRC.

## Introduction

Few groups of animals exhibit more extremes in lifestyle than social insects. Common characteristics of social Hymenoptera include the presence of one or more breeding queen(s) and sterile helpers who assist with reproduction, but beyond these traits, they exhibit remarkable variation. Although they are thought to have originally been monogamous^1^, queen numbers vary among species, representing both monogyny and polygyny and annual and perennial life histories. Colony size also varies, in some species consisting of a queen and a few workers, and others consisting of hundreds of queens and tens of thousands of workers^2^.

**Figure 1:**
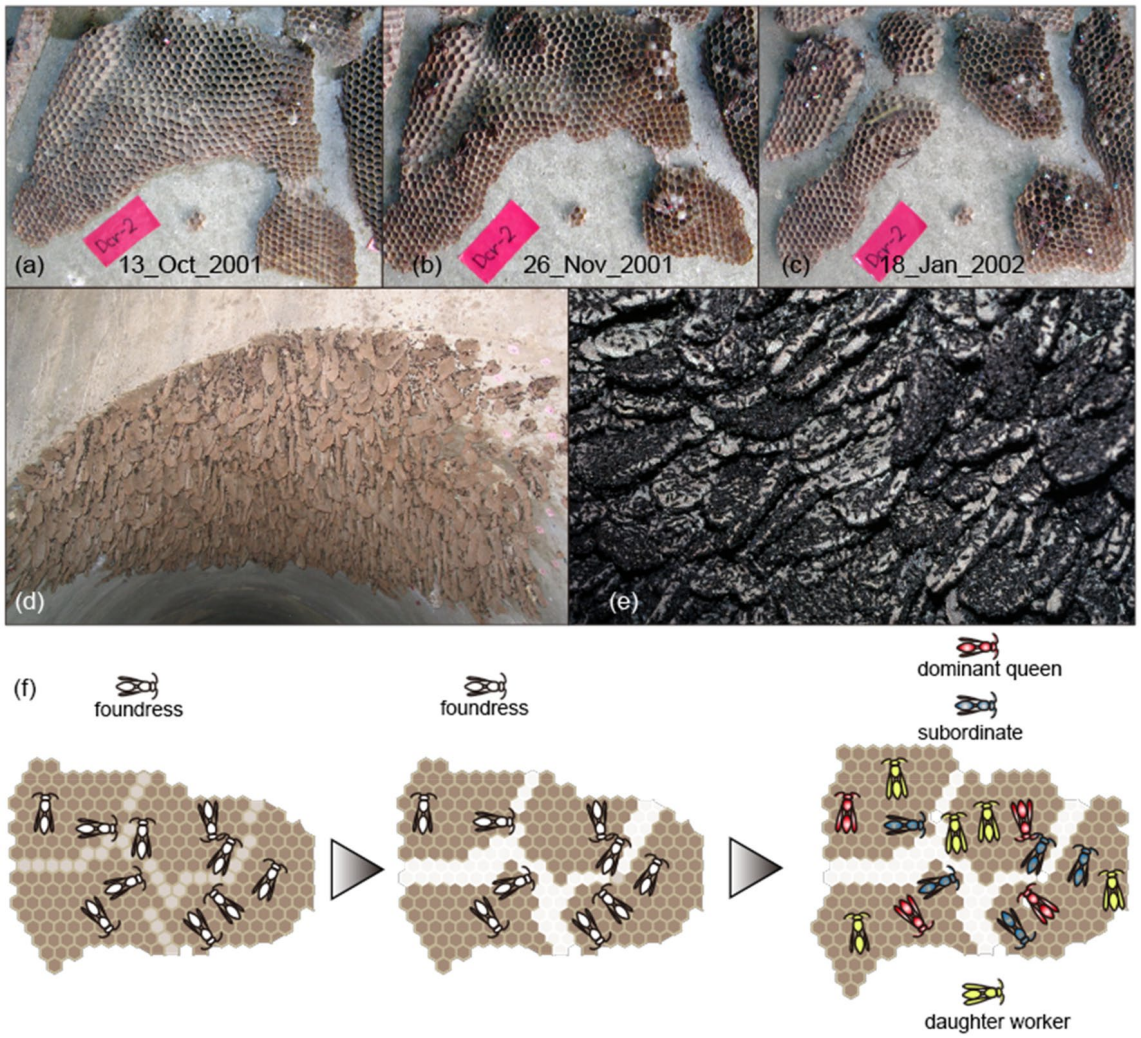
The processes of comb-cutting (**a–c**) and colony aggregation consisting of a group of colonies (**d,e**). Founding stage (October 2001) (**a**), founding stage (November 2001) (**b**), and after worker emergence (post-worker stage; January 2002) at Dinner Creek (**c**). Colony aggregation in late fall (May 2005) (**d**), colony aggregation at the reproductive-producing stage (March 2002) (**e**). Illustration (**f**) shows, from left to right, the process of comb-cutting from the founding stage to the post-worker stage. The location of Dinner Creek was shown in Fig. S4. Photographs courtesy of KT and JK.

The Australian paper wasp *Ropalidia plebeiana* falls at one extreme of population structure owing to its exceedingly dense colony aggregations consisting of a group of colonies (Fig. 1). Each colony consists of a single comb (nesting substrate), and the distances between each colony are very small (< 1 cm). Each colony is independent and is defended against neighboring colonies by colony members^3^, even at distances so small that individuals can touch neighboring combs by their mouths and legs. In addition, the colony aggregations sometimes consist of several thousand individual combs suspended side by side^3^. These large aggregations persist for years^4^; nonetheless, each colony ends each colony season (annual life cycle). Richards^5^ in 1978 described *R. plebeiana* colony aggregations in Cabbage Tree Creek in New South Wales; these aggregations were still extant as of 2018.

Another unique life history of the wasp is that the most combs are reused by inseminated females (foundresses) the following season, as old combs are divided into several new combs (comb-cutting^6^; Fig. 1f) from which new, independent colonies are formed. This is a novel type of colony fission by a group of foundresses and is different from swarm-founding, which is the colony fission by a group of queens and workers in perennial social wasps^2^. In *R. plebeiana*, overwintered foundress groups start their colonies (founding stage) in spring (September). Comb-cutting is frequently observed from the beginning of the colony cycle from spring (October) to the first worker emergence (January; post-worker stage) in which queens and workers co-exist. Upon comb-cutting, each old colony, consisting of a single old comb, is divided into several new colonies, each of which comprises a single new comb (Fig. 1a–c,f). During the post-worker stage, a few dominant foundresses usually monopolize colony reproduction, called queens, and subordinate foundresses become workers. At the end of summer (March), new gynes (candidate foundresses of the following spring) and males emerge from colonies (reproductive-producing stage). Only new gynes overwinter following copulation, and each colony member is dissolved in the autumn declining stage. Overwintering occurs at unknown sites.

In contrast to these unique nesting habits, the genetic structure of the colonies remains relatively poorly understood. The colony and population structures of *R. plebeiana* raise the following ecological questions. First, is comb-cutting based on relatedness? Although nepotistic behavior is thought to be adaptive, clear evidence of such behavior among eusocial insects remains lacking. Second, are long-lasting colony aggregations associated with competition among females over resources, e.g., nesting sites? Such competition could be associated with female philopatry and male-biased colony sex ratios.

We sought to determine whether comb-cutting is kin-based and to reveal the genetic structure at both the population and colony levels within unique colony aggregations. Using these data, we hope to shed light on the unique nesting biology of *R. plebeiana*.

## Results

### Population structure

Using Kinship^7^, we determined that 161 of the 309 foundresses collected from 14 aggregations via arbitrary hand-netting were in full-sib relationships (r = 0.75) under haplodiploidy. We removed these data from further analyses of population structure.

Following sequential Bonferroni correction, no significant departures from Hardy–Weinberg equilibrium were detected across the five loci assessed for the remaining 148 individuals. Similarly, no significant linkage disequilibrium or null alleles were detected in the five loci.

Although the mean likelihood (*K*) was largest at *K* = 1 or *K* = 2, and Evanno’s method^8^ estimated nine populations, our STRUCTURE analysis did not detect any genetic structure among the 14 aggregations at *K* = 2 (Supplementary Fig. S1). The global fixation index (*F*_*ST*_) in the microsatellite dataset was 0.007 (*P* = 0.077), whereas the inbreeding coefficient (*F*_*IS*_) was 0.038 (*P* = 0.042), and the overall fixation index (*F*_*IT*_) was 0.045 (*P* = 0.018), implying no apparent subdivision in the population, but indicating a slight trend toward inbreeding.

We found 18 haplotypes of the cytochrome oxidase subunit I (COI) (accession nos. LC533797–LC533806 and LC554438–LC554447). The global *Ф*_*ST*_ fixation index of this dataset was 0.126 (*P* = 0.001), indicating apparent population subdivision. In the absence of sex-biased gene flow, the expected relationship between microsatellites (represented by *F*_*ST*_) and COI (*Ф*_*ST*_) is as follows: *Φ*_*ST*_ = 4*F*_*ST*_/(1 + 3*F*_*ST*_)^9^. Both *F*_*ST*_ and *Ф*_*ST*_ are known to be influenced by expected heterozygosity (*Hs*)^10^; we, therefore, calculated *G’*_*ST*_ fixation index following Hedrick^11^ and inserted it into the equation. The calculated value of *G’*_*ST*_ was 0.027 (Supplementary Table S1), yielding an expected *Ф’*_*ST*_ of 0.100. This value was lower than the observed *Ф’*_*ST*_ (0.195), indicating female philopatry.

IBD (Isolation By Distance) relationships (Supplementary Fig. S2) were present in the microsatellite data (Mantel test, *r*_*s*_ = 0.425, *P* = 0.009) but not in the COI data (*r*_*s*_ = 0.047, *P* = 0.328). These results indicate that gene flow and drift are balanced in microsatellites, whereas drift is more prominent than gene flow in mitochondrial DNA (mtDNA). We can thus conclude that the population structure of females is more subdivided than that of males.

**Figure 2:**
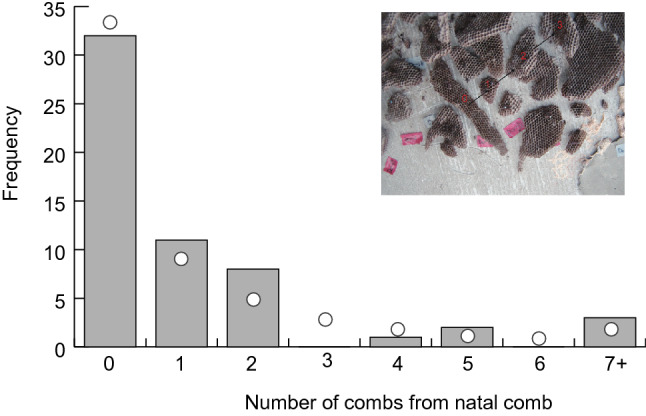
The number of individuals marked in the previous season that was re-sighted the following spring. The inset explains how to count the number of combs starting from the natal comb: 0 indicates a natal comb, and 3 indicates the third comb from the natal comb on which a focal foundress was re-sighted the following spring. We counted the smallest number of combs between the natal comb and the comb where the individual was re-sighted; in this case, three. The frequency distribution did not differ from a negative binomial distribution, representing a contagious distribution model.

Prior to the onset of comb-cutting in spring 2002, we re-sighted 59 foundresses that were marked during the previous season. We counted the number of combs between the natal comb and the comb on which each foundress was re-sighted (Fig. 2). More than half the foundresses settled on natal combs (57.9%; 32/57), indicating female philopatry. The frequency distribution did not differ from a negative binomial distribution (χ^2^_cal_ = 3.458, *df* = 1, *P* = 0.063), which is an expected contagious distribution.

### Colony structure

**Figure 3:**
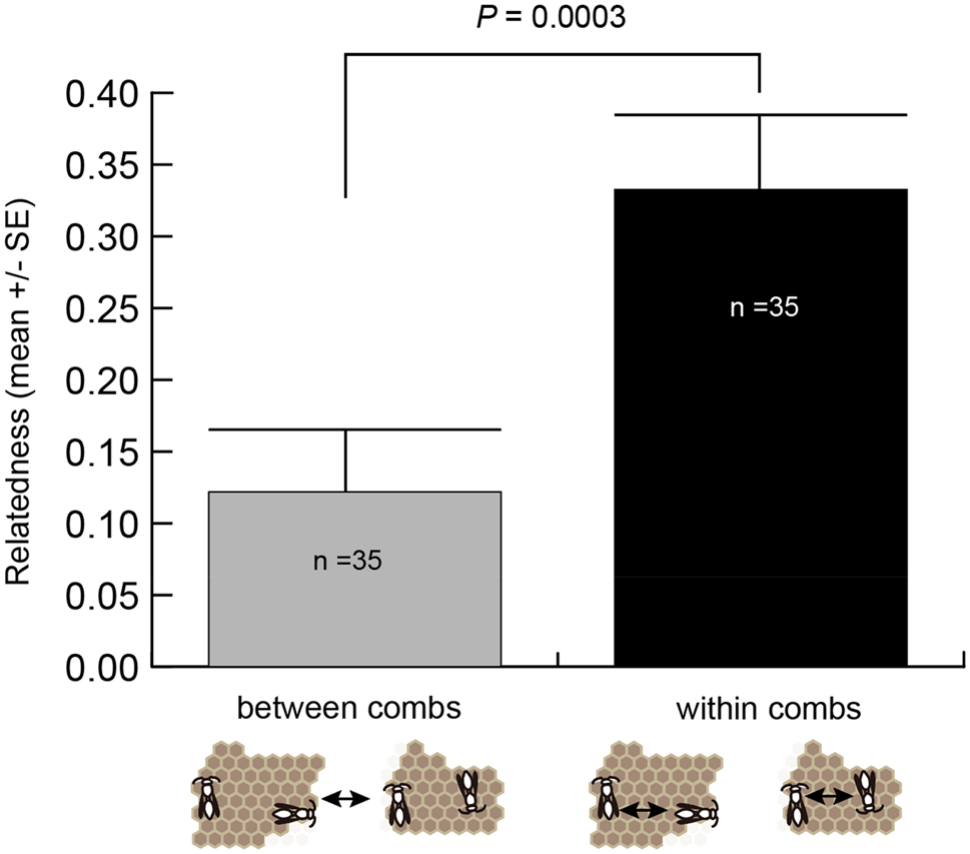
Genetic relatedness among foundresses between and within new combs, who settled on the same old combs prior to cutting. Relatedness values differed significantly (paired t-test, *P* = 0.0003). Each arrow indicated the relationships for which each relatedness was calculated.

We observed the colony founding process, in which each of 16 colonies was divided into two or three new colonies by comb-cutting, yielding a total of 37 new colonies. The mean number of foundresses per colony at pre-comb-cutting was 16.875 ± 2.634 (n = 16) and that at post-comb-cutting was 7.297 ± 0.924 (n = 37), respectively (Table 1). We calculated the following two genetic relatedness values of foundresses, between and within new combs, that settled on the same old combs prior to cutting (Fig. 3). If comb-cuttings were conducted independently by the kin relationship among foundresses, we would expect no significant difference between the two relatedness values. Before the calculation, we removed the data of one colony (DC73) because this colony contained a new comb with one foundress after comb-cutting, and we could not calculate the relatedness within combs. The relatedness within combs was 0.326 ± 0.096 (n = 35), versus 0.107 ± 0.057 (n = 35) between combs, indicating a significant difference in relatedness (paired t-test, *t*_*cal*_ = 3.979, *df* = 34, *P* = 0.0003). The frequency distribution of the relatedness within combs and between combs (Supplementary Fig. S3) indicated that the distribution of the relatedness within combs contained more foundresses with full-sib (r = 0.75) and half-sib (r = 0.375) and fewer foundresses with a zero relatedness than that between combs. These results suggest that foundresses form a new comb with more related individuals than those with less related ones from pre- to post-comb-cutting.

**Table 1:** The number of foundresses at pre-comb-cutting and those at post-comb-cutting for 16 original colonies of *Roplalidia plebeiana*.

Colony code	No. of foundresses at pre-comb-cutting	No. of foundresses at post-comb-cutting	No. of combs after comb-cutting
DC01	23	17 + 6	2
DC02	8	3 + 5	2
DC04	6	2 + 2 + 2	3
DC07	28	19 + 9	2
DC10	9	7 + 2	2
DC23	35	2 + 22 + 11	3
DC24	9	5 + 4	2
DC27	31	16 + 15	2
DC33	31	12 + 19	2
DC66	11	5 + 6	2
DC73^a^	3	2 + 1	2
DC76	23	9 + 6 + 8	3
DC79	6	2 + 4	2
DC83	20	4 + 8 + 8	3
R2	9	5 + 2 + 2	3
R11	18	9 + 9	2
Average ± SE	16.875 ± 2.634	7.297 ± 0.924	2.312 ± 0.120

Each colony consisting of a single old comb was divided into two or three new combs.

^a^This colony was excluded from the relatedness calculation for within combs and between combs because only one foundress was observed in a new comb in post-comb-cutting.

**Figure 4:**
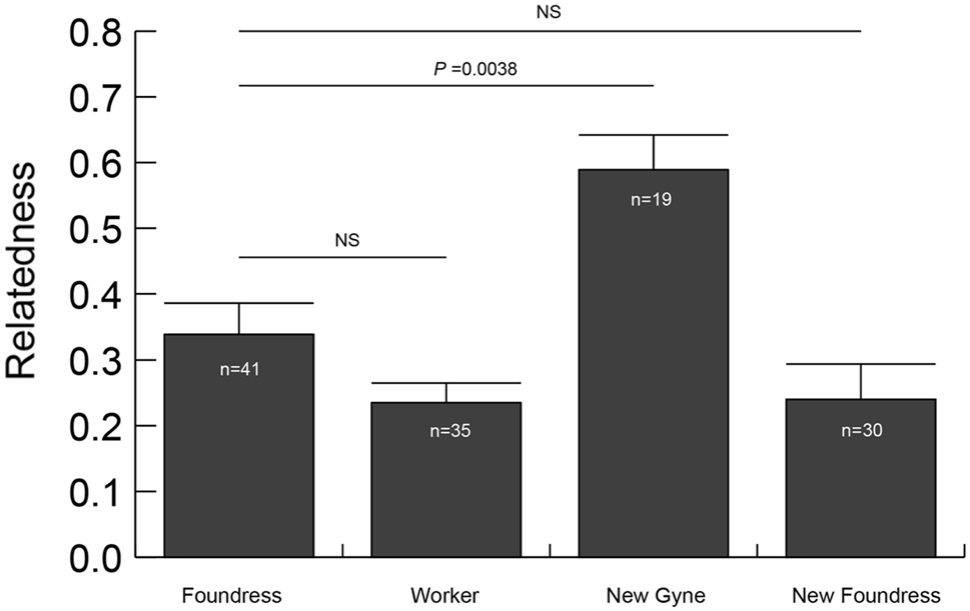
Genetic relatedness among each class of females: foundresses, workers, new gynes, and new foundresses (i.e., foundresses observed the following spring). Welch’s t-test indicated that values among foundresses differed significantly from those among new gynes, even after sequential Bonferroni correction. Sample sizes indicate the number of colonies used to calculate genetic relatedness. *NS* nonsignificant (Welch’s t-test) after sequential Bonferroni correction.

Relatedness was significantly higher among new gynes (0.589 ± 0.053) than among foundresses (0.339 ± 0.047; Welch's t-test and sequential Bonferroni correction, *P* = 0.0038; Fig. 4). However, relatedness among foundresses did not differ from that among workers (0.235 ± 0.030; Welch's t-test, *P* = 0.033) or among new foundresses in the following spring (0.240 ± 0.054; Welch's t-test, *P* = 0.098) following sequential Bonferroni correction.

**Figure 5:**
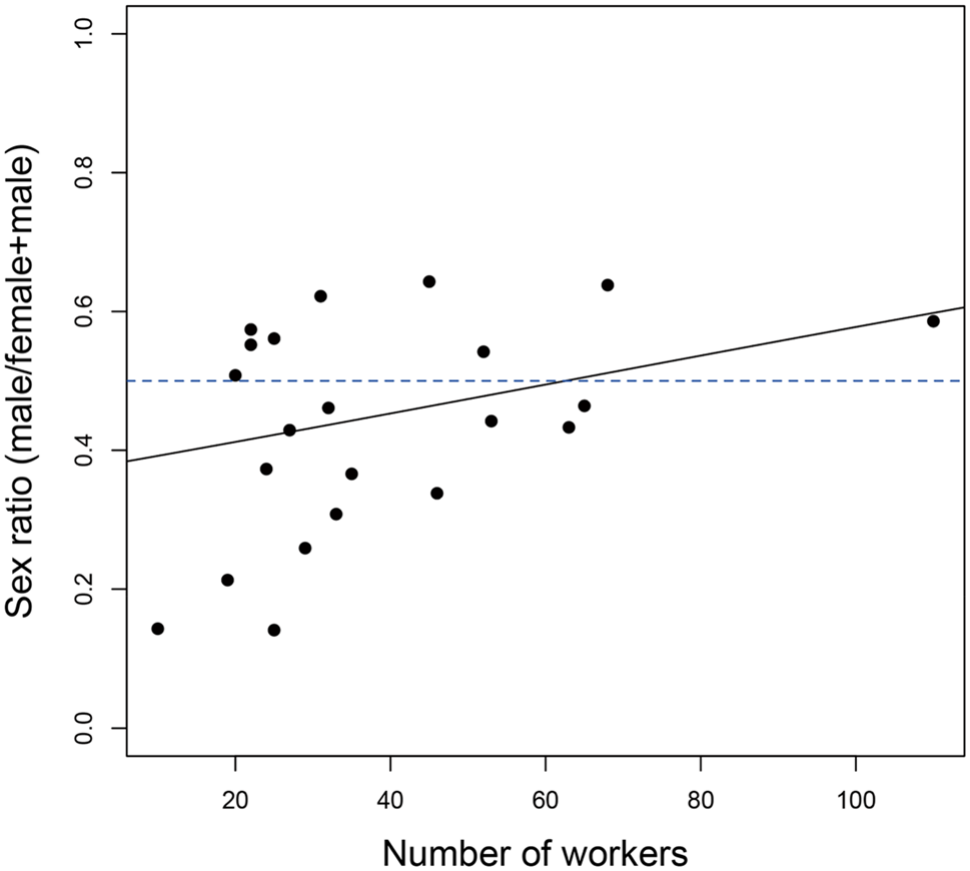
The relationship between the number of workers and sex ratios (proportion of males). The GLM indicated significant differences (see also Tables S2).

We used a GLM with a binomial distribution to analyze the effects of the number and relatedness of workers on the colony sex ratio (Supplementary Tables S2). The number of workers had a positive effect on the sex ratio: colonies with more workers generally were more likely to have male-biased sex ratios (Fig. 5). The mean colony sex ratio was slightly biased toward females (0.443 ± 0.033, n = 24), and the observed population sex ratio was 0.480. Because the dry weight of new gynes was higher than that of males (new gynes: 10.510 ± 0.263 mg, n = 20; males: 9.705 ± 0.292 mg, n = 20), the population investment ratio became 0.460.

We also used a GLM with a Poisson distribution to analyze the effects of the number of foundresses in spring and the number of workers on the total productivity of reproductive individuals (new gynes + males) (Supplementary Table S3). Both had positive effects on total productivity, implying that colonies with more foundresses are more productive.

## Discussion

The paper wasp *R*. *plebeiana* constructs dense colony aggregations at the same sites over time^3,5,6,12^. The combs within these aggregations are reused the following season^3^. The species exhibits an annual cycle in which most colony members disappear in the fall and only the inseminated females (foundresses) overwinter. Although their wintering sites are unknown, most foundresses return to the same colony aggregation sites the following spring^4^.

The estimated global standardized *Ф’*_*ST*_ for mtDNA was higher than the standardized *G’*_*ST*_ for microsatellites, even when values were corrected based on differences in the effective population size between the two markers. In contrast, we observed a weaker population structure in the microsatellite data (Supplementary Fig. S2), suggesting that gene flow frequently occurs in nuclear genes but is limited in maternal mtDNA genes. This implies that new foundresses disperse less than males upon mating, which aligns with the result of our mark–release–recapture study (Fig. 2). The ability of overwintering foundresses to identify their natal combs is particularly interesting. Most foundresses leave the combs and overwinter in unknown sites, and very few individuals overwinter behind the combs^4^. At the onset of spring, they settle either on their natal combs or on a nearby comb. Although the underlying mechanism is not understood, the change in relatedness between foundresses and new gynes suggests that approximately 60% (0.339/0.589) of foundresses start their colonies with their nestmates from the previous year. This value corresponds closely to the frequency of foundresses observed to settle on their natal combs (57.9%; 32/57). Therefore, this might indicate the presence of a relatedness-based threshold for comb-cutting, i.e., the comb is cut when a foundress group comprises < 60% natal nestmates.

The foundresses of *R*. *plebeiana* reuse old combs from the previous season, and those combs are often gnawed into several separate pieces by them^5^. These observations led to hypothesize that each comb is nepotistically divided into smaller new combs and that foundress groups are divided into smaller groups based on kin relationships. Indeed, we found that genetic relatedness among foundresses is higher within combs than between combs (Fig. 3 and Supplementary Fig. S3). Although inclusive fitness theory favors nepotistic behavior, it is observed less frequently than expected in eusocial insects^13^, except for a case in the ant *Formica fusca*^14^, which is likely to be because the fine-scale recognition cue is costly to maintain. In *Polistes dominula*, a significant fraction of wasps chose colonies that did not contain sisters, despite the presence of sisters in nearby colonies^15^. We observed an increase in the relatedness of foundresses following comb-cutting (Fig. 3), suggesting that foundresses recognize their kin upon comb-cutting and new group formation. The search for natal combs in this species may rely on the search for colony aggregation sites, the search for natal combs within these aggregations, and the search for natal nestmates. These processes may require some visual and chemical cues and concomitant long-lasting memories. Each of them may allow for some degree of recognition error, and this may cause the formation of foundress groups containing the relationship with a zero relatedness.

The genetic relatedness of workers was 0.235, and the effective number of mothers for workers was 3.94 based on the following equation if queen’s monandry is a rule:

$${\text{R}}_{{\text{d}}} \, = \,{3}/{4} {\text{N}}\, + \,{\text{R}}_{{\text{q}}} /{4}({1}{-}{1}/{\text{N}}).$$

where R_d_ is the relatedness among daughters, R_q_ is the relatedness among foundresses, and N is the  effective number of mothers^16^. However, the genetic relatedness among new gynes was 0.589, and the number of mothers for new gynes was 1.30. This indicates that colonies of *R*. *plebeiana* are nearly monogynous (or oligogynous), but some subordinate foundresses may be allowed to reproduce in some colonies.

The frequency distribution of the relatedness among foundresses within combs indicated that several combs exhibit zero relatedness (Supplementary Fig. S3). Such non-kin helpers can achieve sufficient fitness if they can inherit colonies^17,18^. However, the opportunity for subordinates to inherit colonies might be quite limited in *R*. *plebeiana*, as we did not observe any queen turnover at our study site. Another possibility is that the fate of the foundress is determined in a pre-imaginal fashion, in which the future queen is determined prior to the death of the existing queen. Her nestmates recognize her as the future queen, and the pheromonal control by the queen over the colony is more important than physical aggression^19,20^. In *R*. *plebeiana*, future queens among foundresses are docile during the founding stage and never forage (Tsuchida, unpublished data), implying that future queens may also signal their presence via pheromones. Such possibilities should be examined in future chemical studies.

We found evidence of female philopatry, which is a prerequisite condition for Local Resource Competition (LRC). The sex ratio became significantly male-biased in larger colonies (Fig. 5). The constant female hypothesis in LRC predicts that colony sex ratios become increasingly male-biased when competition among females over access to resources is prevalent^21,22^. However, though significant, this relationship was moderate, and we did not observe sex ratios that were strongly male-biased in larger colonies, as predicted under LRC. This suggests that other factors counteract LRC in these populations.

One factor that might mitigate LRC is Local Resource Enhancement (LRE), which comprises positive interactions among cooperative breeding females^23–25^. We found a cooperative breeding advantage in *R*. *plebeiana*: the number of foundresses in spring had a positive effect on total productivity (Supplementary Table S3), i.e., colonies with more foundresses were more productive. Cooperative nest founding (polygyny) is more advantageous than solitary founding (monogyny), as polygyny results in greater productivity and more effective defense against parasites compared to monogyny^26–29^. LRE counteracts LRC in *R*. *plebeiana* and, as such, we could not observe sex ratios that were strongly male-biased in the larger colonies.

Another factor that may mitigate LRC is caste plasticity^30,31^, such that protogyny is associated with a moderately female-biased sex ratio. The caste plasticity hypothesis proposes that females are produced before males (protogynous production) whenever there is uncertainty regarding the optimal time to begin producing reproductive individuals. Thus, we might expect that the sex ratio will be female-biased because females may become either workers or new gynes (future queens) until late in their development and respond more flexibly than males^30^. In this study, the reproductive timing of *R. plebeiana* was protogynous (the average date of emergence of reproductive females was February 21 versus March 2 for males), and this might have also counteracted the male-biased sex ratios favored under LRC.

We found that colony fission via comb-cutting is kinship-based. Furthermore, we detected female philopatry in both the mark–release–recapture and genetic studies. We thus conclude that the kinship structure of this species is such that the decline in kinship during the founding stage, assuming that foundresses form groups randomly, is mitigated by female philopatry and kin recognition and subsequently increases due to reproductive skewness among foundresses. This cycle is repeated each season. Our results imply that overwintering foundresses may retain some natal nest-related information during the overwintering period. This ability to discriminate relatives among foundresses is likely to prevent the degree of relatedness within a colony from declining significantly. In addition, we found that sex ratios become more male-biased as colony size increases. However, LRC, which leads to a male-biased sex ratio, may counteract LRE, which leads to a female-biased sex ratio, thus evening out the population sex ratio. In general, no worker control of the sex ratio has been confirmed among primitively eusocial paper wasps^32^. The relatively short annual colony cycle in such wasps may constrain worker control of the sex ratio; colony maintenance is a stronger driver than reproduction, mainly because, unlike perennial species, they have only a single opportunity^33^. Such seasonal constraints may not allow for the evolution of the adaptive traits predicted by theory.

## Methods

### Colony census

We censused 26 colonies from an aggregation at Dinner Creek in New South Wales, Australia (Supplementary Table S4). These routinely censused colonies (RCCs) were censused approximately every three days between mid-October 2001 and late March 2002, and newly emerged adult females from each RCC were individually marked with paint. Females that emerged after February 21, 2002, were designated as new gynes for the following reason: the mean date of emergence of the first new, marked gyne from 19 colonies was February 21, 2002, and we observed 59 marked, overwintered foundresses on September 16, 2002. Newly emerged males were collected with forceps and immediately stored in 99% ethanol (EtOH). The mean date of emergence of the first male among the 24 colonies was March 2. The original queens, if still alive, were collected in the same manner in March and stored in 99% EtOH. Workers that emerged prior to February 21 were collected in March. At the end of March, we collected the entirety of each RCC and counted the number of pupae. We then collected all pupae from each RCC and stored them in 99% EtOH. We observed all collected insects and sexed them based on morphological differences in antennae. We estimated the sex ratio of reproductive individuals (males/[males + new gynes]) for each colony based on these data. We also measured the dry weight of adult males and new gynes to calculate the investment ratio of reproductive individuals.

In addition, we used paint to individually mark 960 foundresses from 111 colonies prior to worker emergence. Between October 2001 and February 2002, we arbitrarily collected colonies, including all colony members (marked foundresses and non-marked, emerged workers), and stored them in 99% EtOH. After each round of collection, we visually inspected whether the old combs had been cut. We successfully monitored this process for 16 combs with 16 foundress groups (n = 269 foundresses). Each of the 16 combs was divided into two or three new combs during the founding stage, and, in total, 37 combs were constructed by 37 foundress groups.

### Sample collection for population structure analysis

We used insect nets to collect 14–40 adults from each of 14 aggregations (n = 309) from March to September 2004, including the Dinner Creek aggregation (Supplementary Fig. S4), and stored them in 99% EtOH.

### Genetic analyses

We extracted DNA from the excised legs of adults and pupae using Chelex 100 (Bio-Rad Laboratories) as described by Walsh et al.^34^. Each DNA solution was stored in TE buffer at 4 °C.

Polymerase chain reaction (PCR) was performed using 1 µL genomic DNA (approximately 1 ng) diluted in a mixture consisting of 1 µL of primer mix (2.5 µM), 0.1 µL of 10 mM dNTP mix, 0.05 µL of Taq polymerase (5 units/µL, Ex Taq; TaKaRa, Kusatsu, Shiga, Japan), 1 µL of 10 × buffer (provided with the polymerase and containing 1.5 mM MgCl_2_), and 6.85 µL of distilled water, yielding a total volume of 10 µL. PCR was performed using a thermal cycler (Gene Amp PCR System 2700; Applied Biosystems, Waltham, MA, USA). After denaturation for 4 min. at 94 °C, the samples were subjected to 30–35 cycles of 1 min. at 94 °C, 1 min. at annealing temperatures appropriate for each primer pair, and 45 s. at 72 °C, with a final extension for 7 min. at 72 °C. We used five primer pairs (Supplementary Table S5). The products were electrophoresed in 8% polyacrylamide gel and visualized using silver staining^35^.

Part of the COI gene was amplified, and the 472 bp sequence was determined using a 3100 DNA sequencer (Perkin-Elmer, Waltham, MA, USA) and a Big-Dye Terminator Cycle Sequence Kit (Applied Biosystems). PCR fragments were sequenced in both directions to ensure accuracy.

### Population structure

For the microsatellite dataset, we checked for null alleles across loci using MICRO-CHECKER^36^ based on the genotype data for the 309 adults. Departures from Hardy–Weinberg assumptions were calculated using GenAlEx ver. 6^37^. Linkage disequilibrium was tested using Genepop ver. 1.2^38^. The 14 aggregations were sampled using arbitrary hand netting. Using Kinship^7^, we identified full-sib relationships and removed them from our population data.

For both the microsatellite and mtDNA datasets, we quantified genetic variation between hierarchical levels in each aggregation and calculated their fixation indices using analysis of molecular variance in Genodive ver. 3.0^39^. This method assesses mtDNA haplotype differentiation based on an analog of the F statistic known as the Ф statistic^40^. F_ST_ (for microsatellites) and Ф_ST_ (for mtDNA) indicate the overall genetic variation among populations and imply sex-biased dispersal patterns. We evaluated genetic differentiation among aggregations using pairwise F_ST_ with 1000 permutations in GenoDive. Significant correlations between genetic differentiation (based on *F*_ST_/(1 – *F*_ST_) values for both markers) and the corresponding geographic straight-line distance (in km) were assessed using a Mantel test with 1000 permutations.

We applied Bayesian clustering in STRUCTURE ver. 2.0^41^ as an alternative measure of the degree of genetic differentiation, using the microsatellite data to delineate the number of genetically identified clusters (*K*) and assign individuals to clusters without using prior information about their locality of origin. We calculated an ad hoc criterion of Δ*K*^8^ to determine optimal *K* values between 1 (panmixia) and 10 and performed 10 runs for each *K* value using 1,000,000 Markov chain Monte Carlo iterations with a burn-in period of 100,000 iterations. These calculations were conducted using Structure Harvester^42^. The assignment index for each individual in each aggregation was calculated using CLUMPAK^43^.

### Colony structure

We genotyped four classes of females, namely, foundresses, workers, new gynes, and new foundresses from the following season. We did not genotype the subordinate foundresses, which later became workers after comb-cutting. The genotypes of new foundresses were assessed based on females collected on September 15–16, 2002. We calculated coefficients of genetic relatedness and inbreeding using Relatedness ver. 4.2c^44^. We calculated the relatedness among foundresses within combs and between combs after comb-cutting and compared the values with a paired test^45^.

Unless otherwise specified, all statistical analyses were performed in R ver. 3.4.1 (R Core Development Team 2020).

## Supplementary Information

Supplementary Information.

## Data Availability

The datasets analyzed in the current study are available from the corresponding author upon request.
