# Supplementary material for: Nepotistic colony fission in dense colony aggregations of an Australian paper wasp
Source: Sci Rep. 2022 Jul 27;12:12868. doi: 10.1038/s41598-022-17117-y (PMC9329314; doi:10.1038/s41598-022-17117-y)
Supplement: Supplementary file 1 — Supplementary Information. [file 41598_2022_17117_MOESM1_ESM.pdf]

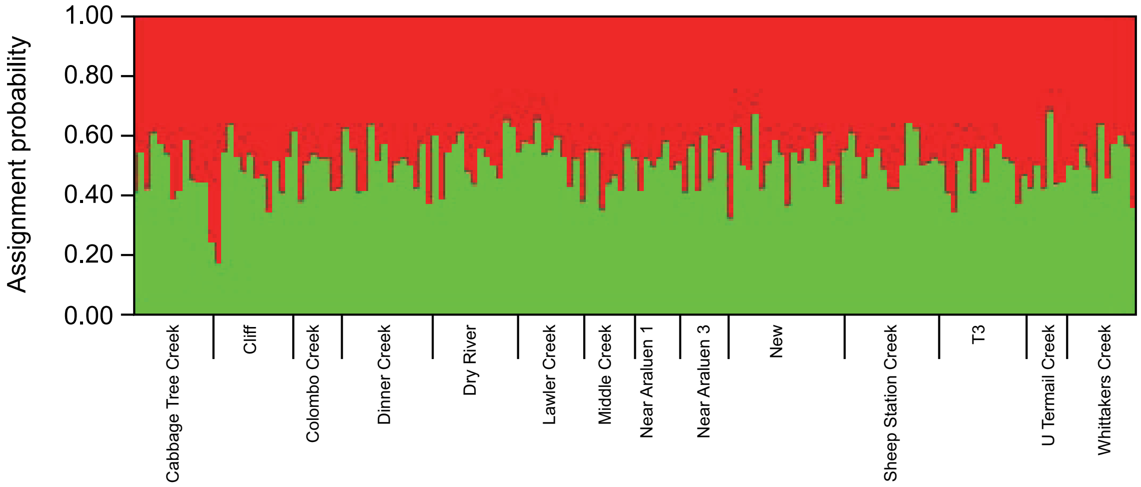

Supplementary Figure S1. Population assignments from STRUCTURE based on microsatellite data. The figure shows $K = 2$ for 148 individuals.

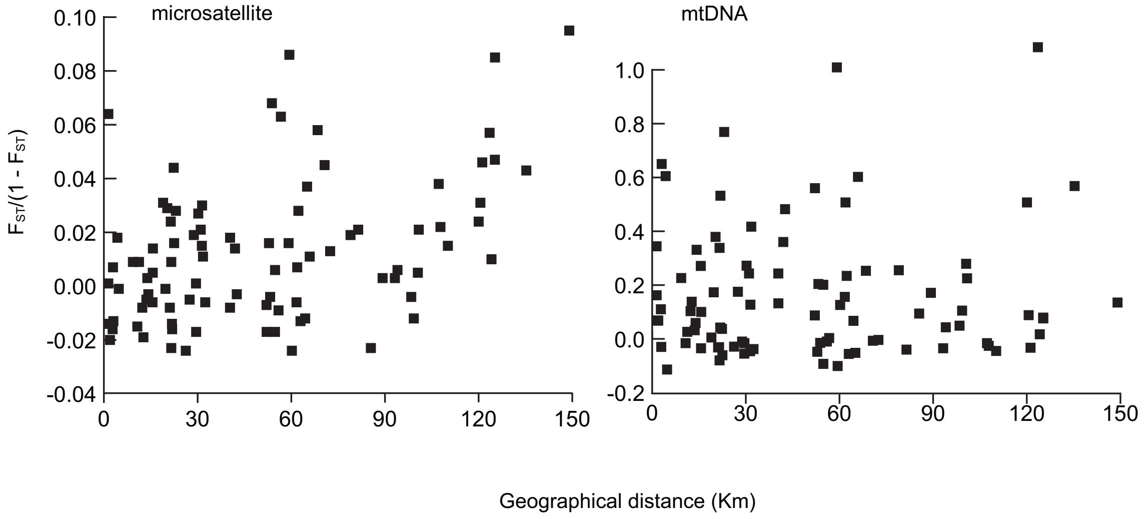

Supplementary Figure S2. Isolation-by-distance relationships based on microsatellite and mtDNA data. A Mantel test indicated that the relationship was significant when using the microsatellite data (r_s = 0.425, P = 0.009) but non-significant when using the mtDNA data (r_s = 0.047, P = 0.328).

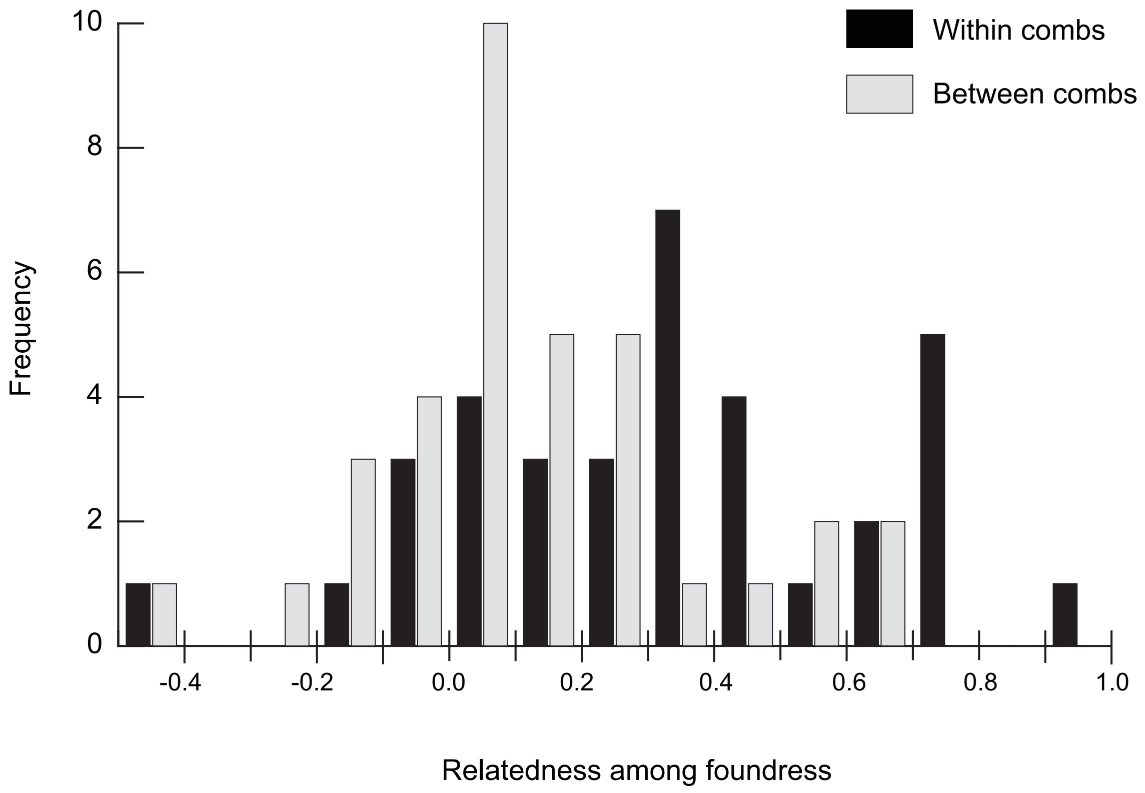

Supplementary Figure S3. Frequency distribution of the relatedness among foundresses within combs and between combs.

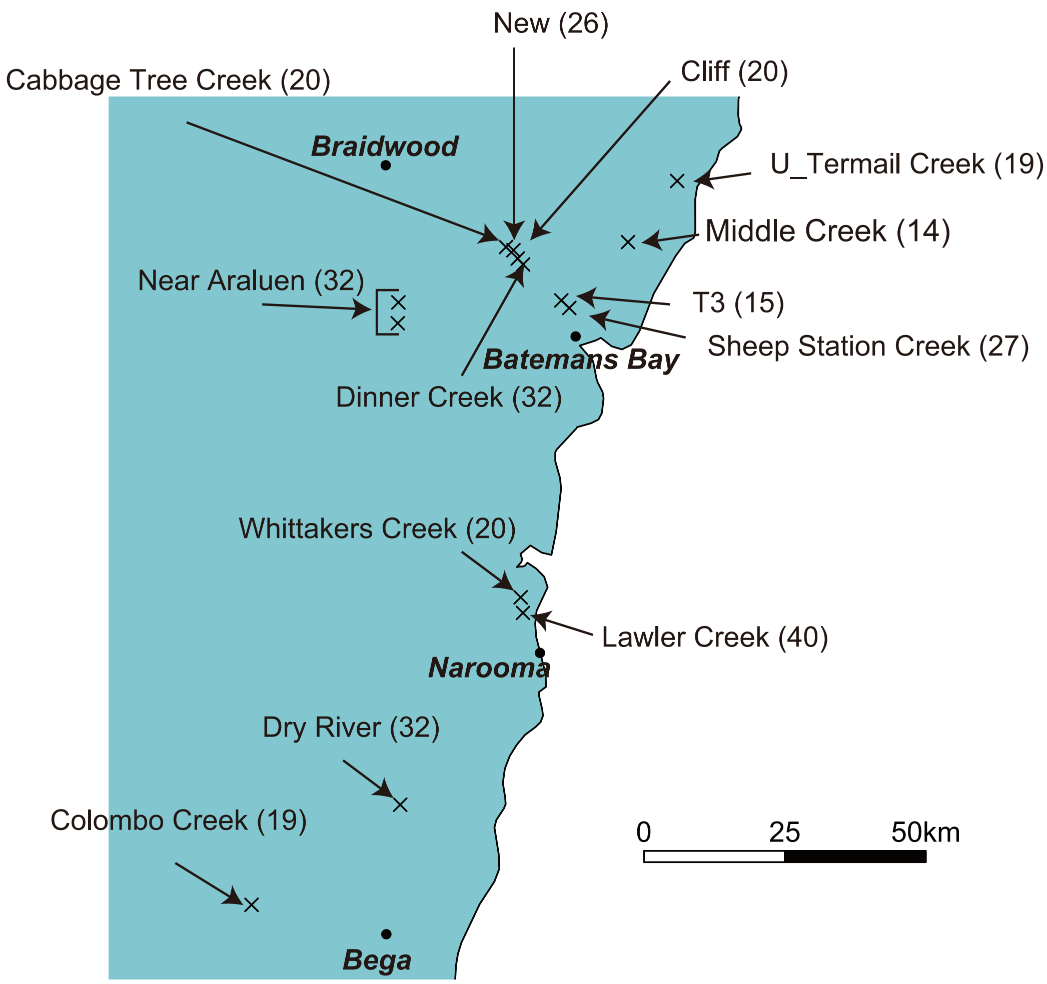

Supplementary Figure S4. Geographical points of 14 aggregations of *Ropalidia plebeiana* in New South Wales, Australia. Numbers in parentheses indicated the number of sampled adult females.

Supplementary Table S1. G'_{ST} (Hedrick) and Φ'_{ST} for all pairs of aggregations in *Ropalidia plebeiana* calculated with microsatellite (upper diagonal, Global G'_{ST} = 0.027^{NS}) and mtDNA (lower diagonal, Global Φ'_{ST} = 0.195***).

Aggregations	(A)	(B)	(C)	(D)	(E)	(F)	(G)	(H)	(I)	(J)	(K)	(L)	(M)	(N)
Cabbage Tree Creek (A)	----	0.017	**0.202**	0.019	0.058	**0.093**	0.025	0.056	-0.027	0.047	-0.018	-0.013	-0.018	0.075
Cliff (B)	0.333	----	**0.104**	-0.037	0.012	-0.029	0.072	0.094	-0.039	-0.030	-0.008	-0.021	0.070	-0.014
Colombo Creek (C)	0.199	0.313	----	0.026	0.027	0.095	0.100	0.030	0.090	**0.122**	0.056	0.074	**0.201**	0.034
Dinner Creek (D)	-0.086	0.176	0.030	----	-0.031	-0.030	-0.002	0.038	-0.067	0.003	-0.051	-0.042	0.039	-0.065
Dry River (E)	0.276	**0.383**	**0.561**	0.165	----	0.044	0.058	0.005	-0.066	-0.010	0.007	0.015	0.112	-0.007
Lawler Creek (F)	-0.155	0.181	-0.080	-0.203	0.181	----	**0.134**	**0.169**	0.011	0.016	-0.043	-0.023	0.046	-0.038
Middle Creek (G)	0.387	**0.482**	**0.643**	0.275	-0.021	0.327	----	0.032	-0.023	0.072	0.013	0.037	0.009	0.029
Near Araluen 1 (H)	-0.124	0.098	-0.430	-0.270	0.191	-0.465	0.360	----	-0.003	0.056	0.060	0.044	**0.148**	**0.140**
Near Araluen 3 (I)	-0.106	0.111	-0.125	-0.300	0.104	-0.369	0.271	-0.554	----	-0.044	-0.018	-0.071	0.042	-0.019
New (J)	**0.416**	**0.510**	**0.667**	**0.314**	0.019	**0.364**	0.000	**0.398**	**0.312**	----	0.023	0.023	0.047	0.038
Sheep Station Creek (K)	0.298	**0.406**	**0.582**	0.191	-0.051	0.241	-0.029	0.269	0.179	0.011	----	-0.055	-0.046	-0.046
T3 (L)	0.055	0.219	0.234	-0.053	0.065	-0.043	0.168	-0.067	-0.101	**0.208**	0.089	----	0.002	-0.012
U Termail Creek (M)	-0.068	0.263	0.349	-0.091	-0.066	-0.082	0.029	-0.046	-0.112	0.072	-0.047	-0.104	----	0.050
Whittakers Creek (N)	**0.568**	**0.523**	-0.101	**0.429**	**0.658**	0.415	**0.721**	-0.005	0.356	**0.738**	**0.673**	**0.506**	**0.566**	----

All pairwise G'_{ST} and Φ'_{ST} with bold letters were significantly positive ($P < 0.05$)

Supplementary Table S2. Generalized linear model with binomial error and logit link function testing the relationship between sex ratio and colony traits (relatedness among workers, number of workers).

Coefficients	Estimate	Std. error	Z value	*P*
Intercept	-0.4676	0.1116	-4.190	***
No. of workers	0.0076	0.0011	6.725	***
Relatedness among workers	-0.0020	0.2682	-0.007	0.99

***: < 0.0001

Supplementary Table S3. Generalized linear model with Poisson and log link function testing the relationship between total productivity (new gynes + male) and colony traits (No. of foundresses in spring and no. of workers).

	Estimate	Std. error	z-value	*P*
Intercept	3.297	0.091	36.205	***
No. of foundresses	0.043	0.113	3.778	***
No. of workers	0.039	0.002	16.662	***
Interaction	-0.001	0.000	-8.114	***

***: < 0.0001

Table S4. Demographic parameters of the routinely censused 26 colonies of *Ropalidia plebeiana* at Dinner Creek.

Colony	No. of males	No. of new gynes	Sex ratio males/(males + new gynes)	No. of workers	No. of foundresses at the founding stage	No. of foundresses at the 1st worker emergence (post-worker stage)	Relatedness among workers
DCR-1	59	69	0.461	32	6	3	0.198
DCR-2 RD	28	77	0.267	32	4	3	0.184
DCR-2 RU	37	30	0.552	22	3	3	0.342
DCR-2 M	2	11	0.154	11	3	2	0.355
DCR-2 LU	51	30	0.630	32	5	4	-0.025
DCR-3	2	9	0.182	8	7	1	----
DCR-4	136	69	0.663	76	9	9	0.252
DCR-5	80	98	0.449	56	9	6	0.467
DCR-6	13	46	0.220	21	10	2	0.267
DCR-7	30	39	0.435	28	6	5	-0.067
DCR-8	46	35	0.568	26	6	2	0.353
DCR-9	109	126	0.464	80	15	9	0.103
DCR-10	67	126	0.347	51	8	6	0.467
DCR-11	263	167	0.612	129	19	16	0.230
DCR-12	71	75	0.486	72	14	7	0.165
DCR-13	11	67	0.141	25	6	2	0.223
DCR-15	28	44	0.389	27	6	4	0.279
DCR-16	66	38	0.635	33	5	2	0.394
DCR-17	128	67	0.656	49	7	6	0.257

DCR-18	53	48	0.525	34	10	4	----
DCR-20	30	28	0.517	21	4	3	0.203
DCR-21	48	79	0.378	39	10	6	0.402
DCR-22	16	33	0.327	36	6	5	0.163
DCR-25	97	74	0.567	60	11	7	0.526
Average	61.29 ± 11.58	61.88 ± 7.80	0.44 ± 0.03	41.67 ± 5.48	7.88 ± 0.80	4.88 ± 0.67	0.26 ± 0.03

*: No. of queens indicated that the number of foundresses survived until 1st February.

**: Harmonic mean

----: We could not calculate the relatedness due to small sample sizes.

Supplementary Table S5. Primer pairs for five loci for microsatellite genotyping for *Ropalidia plebeiana*.

Loci	Forward	Reverse	GenBank Accession No.
RP02	5'-cgagtttcatttaccctcag-3'	5'-aagaactagcgtgtatccca-3'	AB128976
RP04	5'-gtttcaatgggctttagggt-3'	5'-gaacacacatttttggacgc-3'	AB128977
RP06	5'-tacgcgctttgtcgagattc-3'	5'-aagatggggataacgtcggt-3'	AB128978
RP09	5'-gatagagtgtgcgcgaac-3'	5'-tgtgttgtgcgtgagcga-3'	AB128980
Rrev188*	5’-attcaagggctgttctcagtc-3’	5’-gggcgataaagaaaggaaaag-3’	AY247116

*: Henshaw, M. T., Crozier, Y. C. & Crozier, R. H. New microsatellite loci for the socially diverse paper wasp genus *Ropalidia*. *Molecular Ecology Notes*. **3**, 641-643 (2003).
